# Temporomandibular joint inflammation activates glial and immune cells in both the trigeminal ganglia and in the spinal trigeminal nucleus

**DOI:** 10.1186/1744-8069-6-89

**Published:** 2010-12-10

**Authors:** Giovanni Villa, Stefania Ceruti, Matteo Zanardelli, Giulia Magni, Luc Jasmin, Peter T Ohara, Maria P Abbracchio

**Affiliations:** 1 Department of Pharmacological Sciences, Università degli Studi di Milano, via Balzaretti 9, 20133 Milan, Italy; 2 Department of Neurosurgery, Cedars Sinai Medical Center, Los Angeles CA 90013, USA; 3 Department of Anatomy, University of California San Francisco, San Francisco, CA 94143, USA

## Abstract

**Background:**

Glial cells have been shown to directly participate to the genesis and maintenance of chronic pain in both the sensory ganglia and the central nervous system (CNS). Indeed, glial cell activation has been reported in both the dorsal root ganglia and the spinal cord following injury or inflammation of the sciatic nerve, but no data are currently available in animal models of trigeminal sensitization. Therefore, in the present study, we evaluated glial cell activation in the trigeminal-spinal system following injection of the Complete Freund's Adjuvant (CFA) into the temporomandibular joint, which generates inflammatory pain and trigeminal hypersensitivity.

**Results:**

CFA-injected animals showed ipsilateral mechanical allodynia and temporomandibular joint edema, accompanied in the trigeminal ganglion by a strong increase in the number of GFAP-positive satellite glial cells encircling neurons and by the activation of resident macrophages. Seventy-two hours after CFA injection, activated microglial cells were observed in the ipsilateral trigeminal subnucleus caudalis and in the cervical dorsal horn, with a significant up-regulation of Iba1 immunoreactivity, but no signs of reactive astrogliosis were detected in the same areas. Since the purinergic system has been implicated in the activation of microglial cells during neuropathic pain, we have also evaluated the expression of the microglial-specific P2Y_12 _receptor subtype. No upregulation of this receptor was detected following induction of TMJ inflammation, suggesting that any possible role of P2Y_12 _in this paradigm of inflammatory pain does not involve changes in receptor expression.

**Conclusions:**

Our data indicate that specific glial cell populations become activated in both the trigeminal ganglia and the CNS following induction of temporomandibular joint inflammation, and suggest that they might represent innovative targets for controlling pain during trigeminal nerve sensitization.

## Background

Chronic pain is a pathological condition mainly associated with damage or dysfunction of peripheral and central sensory pathways (neuropathic pain), or to tissue inflammation (inflammatory pain) [1]. Despite efforts in the last decades towards the understanding of its pathophysiology and the development of new drugs, chronic pain still remains a difficult to manage and disabling condition. The reason for this failure may be in part due to the fact that most of the available drugs target neurons [2,3], whereas increasing evidence now indicates that glial cells also play an important role in the generation and maintenance of chronic pain [4-7]. During pathological pain states, in the central nervous system (CNS) both microglial cells and astrocytes become activated and start releasing proinflammatory signals which are responsible for the hyperexcitability of nociceptive pathways, thereby leading to the development of hyperalgesia and allodynia [8-11]. Accordingly, the pharmacological inhibition of glial cell function effectively attenuates the development of both neuropathic [12,13] and inflammatory pain [14].

Accumulating evidence suggests that glial cells of sensory ganglia also participate in the development and maintenance of chronic pain conditions [15,16]. Following stimulation of neurons in the dorsal root ganglia (DRG), satellite glial cells (SGCs) wrapped around neuronal bodies [17] become activated and increase neuronal excitability by releasing inflammatory mediators such as tumour necrosis factor-α (TNFα) [18]. Following sciatic nerve injury, an increased expression of interleukin (IL)-6 [19] and of other pro-inflammatory cytokines [20] is also detected in SGCs. Moreover, DRG neurons release chemokines that trigger macrophage invasion, suggesting that the latter cell population is also involved in the regulation of chronic pain [21-23].

The trigeminal ganglion (TG) is the location of primary afferent neurons for sensing and relaying nociceptive sensations associated with painful conditions such as dental pain, trigeminal neuralgia, and temporomandibular disorders [24]. Cutaneous inflammation triggers the activation of SGCs in the TG, leading to the release of the pro-inflammatory cytokine IL-1β [25], which in turn increases the firing activity of nociceptors [26]. Interestingly, the selective silencing of the SGCs proteins connexin 43 or glutamine synthase in the TG significantly reduced the hyperalgesia associated with the chronic constriction of the infra-orbital nerve [27], thus suggesting that SGCs are key determinants of chronic pain intensity. A well-established model of inflammatory pain, which shares several characteristics with migraine-associated TG sensitization [28,29], is based on the injection of pro-inflammatory mediators (e.g., capsaicin or Complete Freund's Adjuvant, CFA) in the temporomandibular joint (TMJ). It was shown that TMJ inflammation potentiates the excitability of nociceptive neurons in both the TG [26] and in the spinal trigeminal nucleus [30], and leads to increased neuron-to-SGC communication within the TG [31,32], but to date, no studies have explored the reaction of glial cells in the whole trigeminal-spinal system following the induction of TMJ inflammation. Therefore, in the present study we have characterized the reaction of peripheral nervous system (PNS) and CNS glial cells to the injection of CFA into the rat TMJ.

## Results

### Development of mechanical allodynia and inflammation after injection of CFA into the TMJ

**Figure 1:**
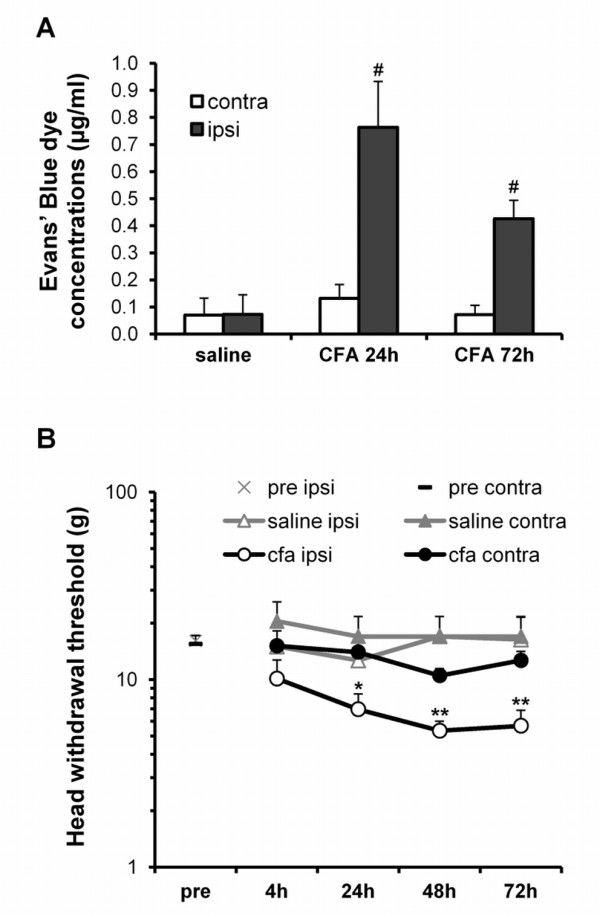
**Injection of CFA into the TMJ produces plasma extravasation and mechanical allodynia**. (A) The development of inflammation was analyzed by injecting the Evans' blue dye through the tail vein. A significantly higher amount of dye was extracted from the ipsilateral TMJs of CFA-injected rats compared to the contralateral side at both 24 h and 72 h p.i. Since no significant differences between saline-injected rats at 24 h and 72 h have been observed, data have been pooled together, and shown here as "saline". (B) Rats were tested for the development of mechanical allodynia by probing the contralateral (contra) and ipsilateral (ipsi) orofacial regions with von Frey filaments before (pre) and post injection of saline or CFA into the TMJ. The head withdrawal threshold force, in grams (g), was measured. Y-axis = log_10 _scale. ** p < 0.01, and * p < 0.05 compared to the contralateral side of CFA-injected rats, # p < 0.01 compared to the contralateral tissue; one-way ANOVA.

First of all, to evaluate inflammation following CFA injection, we measured the TMJ extravasation of the Evans' blue dye injected into the tail vein. While there was no difference in the dye concentration in the TMJs of rats injected with saline (0.07 ± 0.07 μg/ml for the ipsilateral side vs. 0.07 ± 0.06 μg/ml for the contralateral side, p = 0.977; n = 7 animals) (Figure 1A), a significantly greater amount of dye was extracted from the ipsilateral TMJ of CFA-injected rats, both at 24 h p.i. (0.76 ± 0.17 μg/ml vs. 0.13 ± 0.05 μg/ml for ipsi- and contralateral tissue, respectively, p < 0.01; n = 6 animals), and, to a lesser extent, at 72 h p.i. (0.43 ± 0.07 μg/ml vs. 0.07 ± 0.03 μg/ml for ipsi- and contralateral tissue, respectively, p < 0.01; n = 7 animals) (Figure 1A).

The development of allodynia, due to trigeminal sensitization secondary to the primary inflammatory event, was then evaluated by probing the orofacial regions of saline- or CFA-injected animals with von Frey filaments. Baseline values for mechanical threshold were determined in non-inflamed rats. Mean head withdrawal thresholds were measured from the left and right orofacial regions (16.11 ± 2.06 g for the right side and 15.50 ± 1.73 g for the left side; n = 12 animals) (Figure 1B). Rats were then injected with either saline or CFA into the left TMJ and tested for their pain behavior. A significantly lower threshold to innocuous mechanical stimuli was measured for the ipsilateral side of CFA injected rats, starting 24 h post injection (p.i.) (Figure 1B and Table 1). No significant changes between the ipsi- and the contra-lateral mechanical thresholds were observed in control saline-injected animals (Figure 1B and Table 1).

**Table 1:** Mean head withdrawal threshold values after saline or Complete Freund's Adjuvant (CFA) injection in the temporomandibular joint.

Stimulus	Time post injection	Ipsilateral (g)	Contralateral (g)	n° of rats	p value *(ipsi vs. contra)*	p value *(ipsi vs. baseline)*
**baseline**	-	15.5 ± 1.73	16.1 ± 2.06	12	-	-
**saline**	4 h	15.0 ± 0.00	20.5 ± 5.50	4	0.423	0.813
	24 h	12.7 ± 2.33	17.0 ± 4.73	4	0.457	0.395
	48 h	17.0 ± 4.73	17.0 ± 4.73	4	1.000	0.845
	72 h	16.3 ± 5.24	17.0 ± 4.73	4	0.929	0.962
**CFA**	4 h	10.1 ± 2.59	15.1 ± 3.07	8	0.231	0.088
	24 h	6.94 ± 1.45	14.0 ± 2.47	8	< 0.05	< 0.01
	48 h	5.33 ± 0.67	10.5 ± 0.96	8	< 0.01	< 0.01
	72 h	5.67 ± 1.20	12.7 ± 1.48	8	< 0.01	< 0.01

g: grams; ipsi: ipsilateral side; contra: contralateral side.

These results confirm that the injection of CFA into the TMJ induces a persistent local inflammation, associated with the development of mechanical allodynia.

### SGCs and macrophages are selectively activated in trigeminal ganglia following TMJ inflammation

**Figure 2:**
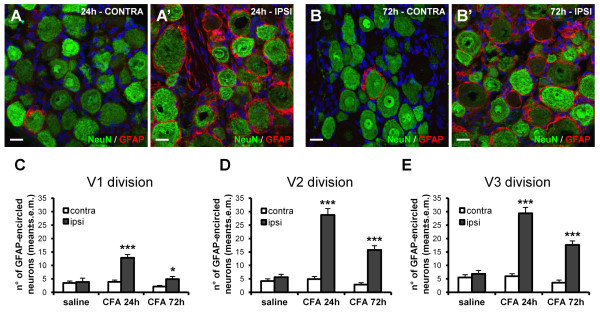
**GFAP immunoreactivity is increased in SGCs following induction of TMJ inflammation**. (A, B) Few GFAP^+ ^SGCs (red) encircling NeuN^+ ^neurons (green) were detected in the contralateral (CONTRA) TG of CFA-injected rats, or in TGs from saline-injected rats (see below). A significant increase in the number of GFAP-encircled neurons was instead observed in the ipsilateral (IPSI) TG starting at 24 h post CFA injection (A', B'). Nuclei were labeled with the Hoechst 33258 dye (blue). Scale bars: 20 μm. (C-E) The number of GFAP-encircled neurons was increased in all 3 divisions (V1-ophthalmic, V2-maxillary, V3-mandibular) of ipsilateral (ipsi) TG. Similar, although smaller, changes were observed at 72 h p.i. Since no significant differences between saline-injected rats at 24 h and 72 h have been observed, data have been pooled together, and shown here as "saline". Data are expressed as number of GFAP-encircled neurons per counting field at 40x magnification, and refer to 2 independent experiments. *** p < 0.001 and * p < 0.05 compared to the contralateral side; one-way ANOVA.

We next evaluated the morphological and biochemical consequences of the induction of TMJ inflammation in the TG, with particular focus on SGCs [33]. Previous studies have reported that either tooth pulp injury or TG inflammation induces SGCs hypertrophy, with increased expression of GFAP [25,34,35], which is considered to be a marker of reactivity and activation for this particular type of glia. In our experimental model, very low levels of GFAP immunoreactivity were observed in the contralateral side of CFA injected rats, as measured by counting the number of TG neurons encircled by GFAP-positive (GFAP^+^) SGCs (Figure 2A, B). As expected [25], a large increase in the number of GFAP-encircled neurons was observed in the ipsilateral TG at both 24 h (Figure 2A') and 72 h p.i. (Figure 2B'). From 24 h p.i. the number of GFAP-encircled neurons was higher in the ipsilateral V3-mandibular (29.38 ± 2.20 in the ipsilateral side vs. 5.99 ± 0.94 in the contralateral side, p < 0.001; n = 7 animals), and V2-maxillary (28.79 ± 2.30 vs. 4.92 ± 1.00, p < 0.001; n = 7 animals) division of the trigeminal nerve. The same effect was also observed in the V1-ophthalmic division, although to a lesser extent (12.83 ± 1.24 vs. 3.88 ± 0.69, p < 0.001; n = 7 animals) (Figure 2C-E). At 72 h p.i. a reduction in GFAP staining was detected compared to the values at 24 h, but it was still significantly higher with respect to the contralateral side or controls (p < 0.05 and p < 0.001 for the V1 and V2-3 division respectively; n = 7 animals) (Figure 2C-E). No differences in the number of GFAP-encircled neurons were found between the ipsi- and contralateral TGs in saline-injected rats (p = 0.805, p = 0.319, p = 0.432 for the V1, V2, V3 division respectively; n = 7 animals) (Figure 2C-E).

**Figure 3:**
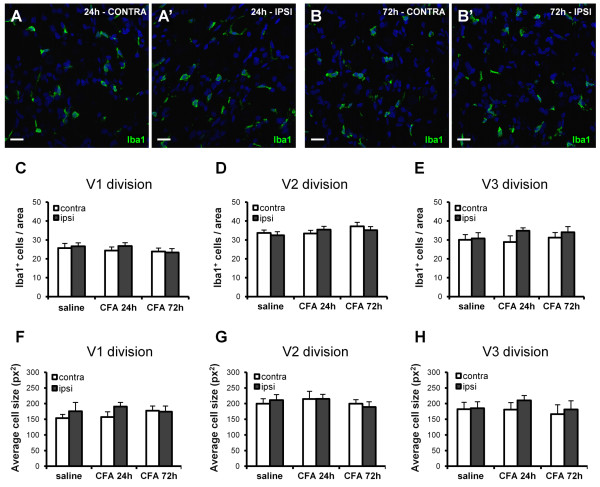
**No changes in the number of resident TG macrophages are induced by injection of CFA in the TMJ**. Twenty-four (A, A') or 72 hours (B, B') after CFA injection, Iba1 immunostaining (green) of contralateral (CONTRA) and ipsilateral (IPSI) TG showed no morphological changes of Iba1^+ ^macrophages. Nuclei were labeled with the Hoechst 33258 dye (blue). Scale bars: 20 μm. No differences in either the number (C-E) or in the average cell size (F-H) of Iba1^+ ^macrophages were detected in any of the three TG divisions in inflamed compared to saline-injected rats. Since no significant differences between saline-injected rats at 24 h and 72 h have been observed, data have been pooled together, and shown here as "saline".

**Figure 4:**
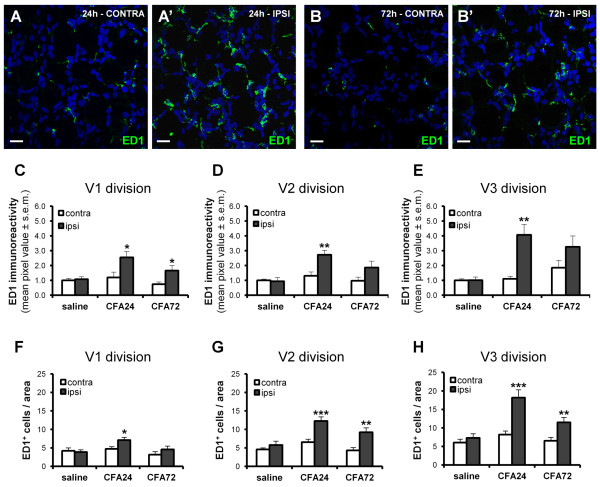
**Injection of CFA in the TMJ activates resident TG macrophages**. (A-B') ED1 immunostaining (green) of activated macrophages in the V3-mandibular TG division, showing upregulation at both 24 h (A, A') and 72 h (B, B') post CFA injection. Nuclei were labeled with the Hoechst 33258 dye (blue). Scale bars: 20 μm. (C-E) Densitometric analysis of ED1 immunoreactivity, showing that, at 24 h post CFA injection, a significant upregulation of ED1 staining was detected in any of the three TG divisions, and that a lower, although not statistically significant, effect was also present at 72 h. (F-H) Results from densitometric analysis were fully confirmed by counting the number of ED1^+ ^cells in an identical area for each coverslip. Since no significant differences between saline-injected rats at 24 h and 72 h have been observed, data have been pooled together, and shown here as "saline". Data are expressed as number of ED1^+ ^macrophages per counting field at 40× magnification, and refer to 3 independent experiments. * p < 0.05, ** p < 0.01, and *** p < 0.001 compared to the contralateral side; one-way ANOVA.

Macrophages have been reported to infiltrate the DRG following sciatic nerve damage or hindpaw inflammation [36,37]. We therefore evaluated whether a similar effect also takes place in the TG after the development of TMJ inflammation. Surprisingly, in any of the three divisions of the ipsilateral TG no changes in the number of Iba1^+ ^resident macrophages and no difference in their mean cell size were observed at either 24 h or 72 h following CFA injection (see Figure 3, panels A-B' for representative images of Iba1 staining, and panels C-E and F-H for quantification of Iba1 cell number and of their average cell size, respectively). Nevertheless, clear signs of macrophagic activation were detected by using an antibody directed against the lysosomal antigen ED1, a marker of activated macrophages (Figure 4A-B') [38-40]. Quantification of results has been performed by both a densitometric analysis of ED1 immunostaining (Figure 4C-E), and by counting the number of ED1^+ ^cells in identical areas in each coverslip (Figure 4F-H). Both analyses demonstrated a significant increase of ED1 immunostaining in the three divisions of ipsilateral TG with respect to the contralateral side at 24 h after CFA injection, with the highest effect detected in the V3-mandibular division (densitometric analysis: 111.39 ± 16.71 pixels for the ipsilateral side vs. 36.44 ± 10.73 pixels for the contralateral side, p < 0.01, n = 6 animals; number of ED1^+ ^cells: 18.19 ± 2.10 cells for the ipsilateral side vs. 8.18 ± 0.96 cells for the contralateral side, p < 0.01; n = 7 animals; Figure 4E, H). These effects persisted at 72 h p.i., although to a lesser extent (Figure 4). These results suggest that, early after induction of TMJ inflammation, there is no recruitment of new inflammatory cells from the bloodstream, but rather a strong activation of local resident macrophages.

### Microglial cells, but not astrocytes, are activated in the spinal trigeminal nucleus following TMJ inflammation

Although previous studies reported that CNS glial cells (astrocyte and microglial cells) become activated following CFA-induced sciatic nerve inflammation [41-43], no studies on the role of these cells after inflammatory sensitization of the TMJ are available.

The primary afferent fibers of the trigeminal nerve terminate in the spinal trigeminal nucleus of the brainstem, which extends through the pons and medulla, and finally overlaps with the dorsal horn of the cervical spinal cord [44]. Moving along its rostro-caudal axis, the spinal trigeminal nucleus can be subdivided in subnucleus oralis, subnucleus interpolaris, and subnucleus caudalis [44].

**Figure 5:**
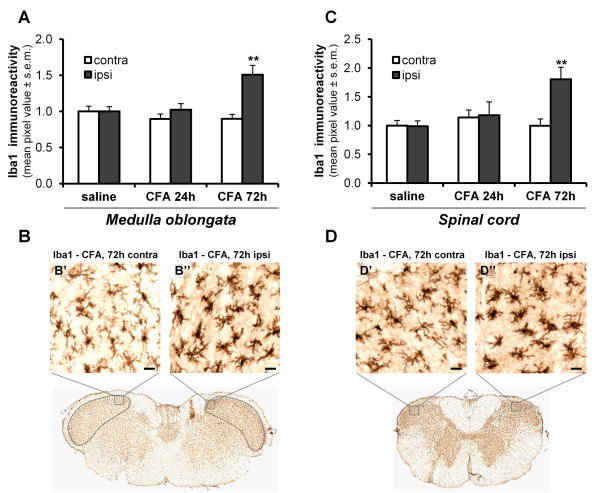
**TMJ inflammation induces microglial activation in the spinal trigeminal nucleus**. (A, C) Densitometric analysis of Iba1 immunoreactivity (number of immuno-positive pixels, see Methods) showing a significant increase in Iba1 immunoreactivity in the ipsilateral side of both the trigeminal subnucleus caudalis in the medulla oblongata (delimited by the dotted line; A) and the dorsal horn of the cervical spinal cord (C) 72 h after CFA injection. The mean values of pixel intensity have been normalized to the values obtained from the contralateral side of saline injected rats, set to 1.0. Since no significant differences between saline-injected rats at 24 h and 72 h have been observed, data have been pooled together, and shown here as "saline". ** p < 0.01 compared to the contralateral side; one-way ANOVA. (B-B'', D-D'') Resting microglial cells (i.e., ramified cells with fine processes) were detected in the contralateral side of the trigeminal subnucleus caudalis (B') and of the cervical dorsal horn (D'), whereas activated microglial cells (i.e. cells with thicker ramifications) were observed ipsilaterally (B'', D''). Scale bars: 20 μm.

Immunoreactivity levels for Iba1 (a marker for microglial cells) and GFAP (a marker for astrocytes) in the different regions of the spinal trigeminal nucleus were thus evaluated in saline and CFA-injected rats. As shown in Figure 5, in saline-injected rats no changes in Iba1 immunoreactivity were observed between the contra- and ipsi-lateral sides of either the trigeminal subnucleus caudalis of the medulla oblongata (p = 0.836; n = 8 animals) (Figure 5A) or of the dorsal horn of the cervical spinal cord (p = 0.918; n = 8 animals) (Figure 5C). Seventy-two hours after CFA administration, Iba1 immunoreactivity was significantly up-regulated in the dorsal laminae of the trigeminal subnucleus caudalis (normalized values: 1.51 ± 0.13 pixels for the ipsilateral side vs. 0.90 ± 0.06 pixels for the contralateral side, p < 0.01; n = 8 animals) (Figure 5A). Moreover, in the ipsilateral side microglial cells displayed shorter and thicker ramifications, a typical characteristic of activated microglia, when compared to the fine processes of microglia in the contralateral side (Figure 5B', B''). Similar changes were also observed in the dorsal horn of the cervical spinal cord (normalized values: 1.80 ± 0.21 pixels for the ipsilateral side vs. 1.00 ± 0.12 pixels for the contralateral side, p < 0.01; n = 7 animals) (Figure 5C, D).

**Figure 6:**
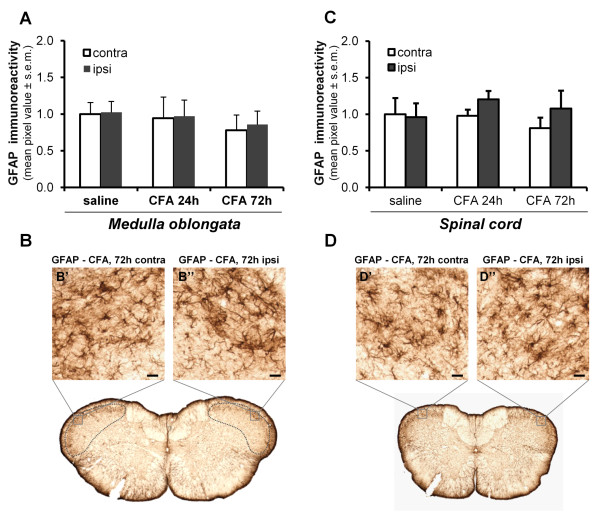
**TMJ inflammation does not affect the activation state of GFAP^+ ^astroglial cells in the CNS**. (A, C) Densitometric quantification of GFAP immunoreactivity in the trigeminal subnucleus caudalis (A) and the cervical dorsal horn (C) revealed no changes between the contralateral (contra) and the ipsilateral (ipsi) sides of CFA inflamed rats. The mean values of pixel intensity have been normalized to the contralateral side of saline injected rats, set to 1.0. Since no significant differences between saline-injected rats at 24 h and 72 h have been observed, data have been pooled together, and shown here as "saline". (B-B'', D-D'') No changes in astroglial cell morphology in the trigeminal subnucleus caudalis (delimited by the dotted line; B-B'') and the cervical dorsal horn (D-D'') were seen. Scale bars: 20 μm.

Conversely, no reactive astrogliosis was detected in the spinal trigeminal nucleus, both in terms of GFAP immunoreactivity (p = 0.933 and p = 0.779 for the trigeminal subnucleus caudalis at 24 h and 72 h respectively; p = 0.156 and p = 0.364 for the cervical dorsal horn at 24 h and 72 h respectively; n = 7 animals) (Figure 6A, C), and of the morphology of GFAP^+ ^astrocytes (Figure 6B, D). We conclude that, during the sub-acute phase of CFA-induced TMJ inflammation, microglial cells, but not astrocytes, are selectively activated in the CNS regions relaying nociceptive information from the TG.

### The purinergic P2Y_12 _receptor is selectively expressed by microglial cells in the CNS, but it is not up-regulated following TMJ inflammation

**Figure 7:**
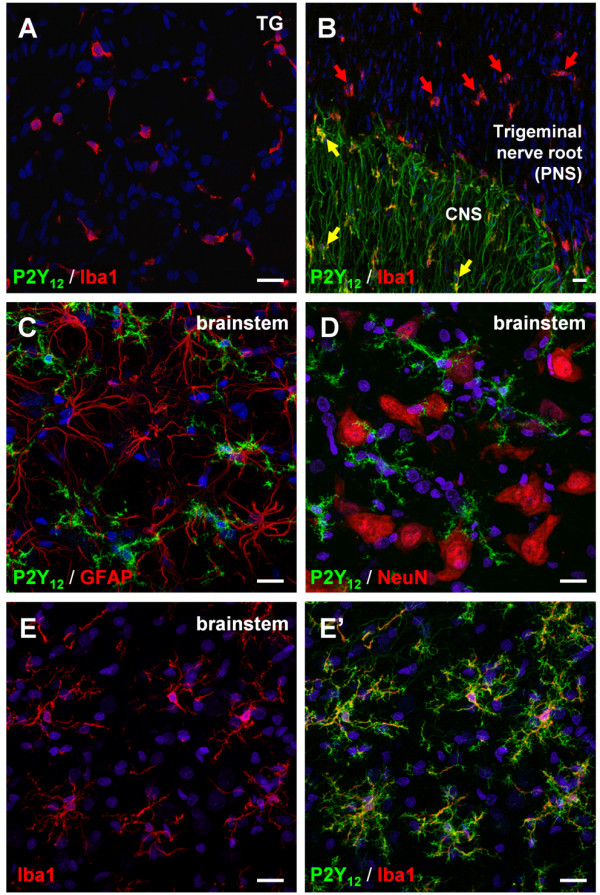
**P2Y_12 _receptor immunoreactivity is absent in the TG, but selectively localized to microglial cells in the brainstem**. (A) In the TG, no staining for the P2Y_12 _receptor subtype (green) was detected in Iba1-expressing macrophages (red). (B) At the trigeminal nerve root (i.e., the PNS/CNS boundary) Iba1^+^/P2Y_12_^+ ^microglial cells were instead observed (yellow arrows). (C-E') In the brainstem, no colocalization between P2Y_12 _receptor (green) and either the astrocytic marker GFAP (red; C), or the neuronal marker NeuN (red; D) was observed. P2Y_12 _receptor immunoreactivity was instead specifically found on Iba1^+ ^microglial cells (red; E, E'). Nuclei were labeled with the Hoechst 33258 dye (blue). Scale bars: 20 μm.

ATP and other extracellular nucleotides participate in pain transmission under both normal and pathological conditions [45-47]. Their receptors (namely the ligand-gated P2X receptors, P2X_1-7_, and the metabotropic G protein-coupled P2Y receptors, P2Y_1,2,4,6,11,12,13,14_) are expressed not only by sensory neurons [48,49], but also by all the different glial cell populations involved in nociception, including SGCs [18,50], macrophages [51], astrocytes [52], and microglia [53]. In particular, in this latter cell population the P2Y_12 _receptor subtype was shown to be up-regulated following nerve injury, and its pharmacological or biotechnological inhibition prevented the development of mechanical allodynia [54,55]. On this basis, we analyzed the possible changes of P2Y_12 _receptor expression in our inflammatory model. Using a specific antibody directed against the C-terminal domain of the rodent P2Y_12 _receptor [56], we were not able to detect any staining within the TG of control animals (Figure 7A). This finding contrasts with previous reports indicating the presence of P2Y_12 _receptor mRNAs in both rat DRGs [57] and mouse TG [50]. Interestingly, in the same tissue sections P2Y_12 _receptor immunoreactivity was detected at the boundary between the trigeminal nerve root (i.e., the PNS) and the CNS (Figure 7B), thus indicating that the expression of this receptor subtype is probably restricted to cells of the CNS. Furthermore, while P2Y_12 _receptor and Iba1 immunostaining was colocalized in CNS microglial cells (Figure 7B, yellow arrows), Iba1^+ ^macrophages resident in the trigeminal nerve root were P2Y_12_-negative (Figure 7A and Figure 7B, red arrows). In the brainstem, no P2Y_12 _receptor expression was observed in either GFAP^+ ^astrocytes (Figure 7C), or NeuN^+ ^neurons (Figure 7D). Indeed, the P2Y_12 _receptor subtype was only seen in Iba1^+ ^microglial cells (Figure 7E, E'), confirming previous reports indicating this receptor expressed by CNS microglia [54-56].

**Figure 8:**
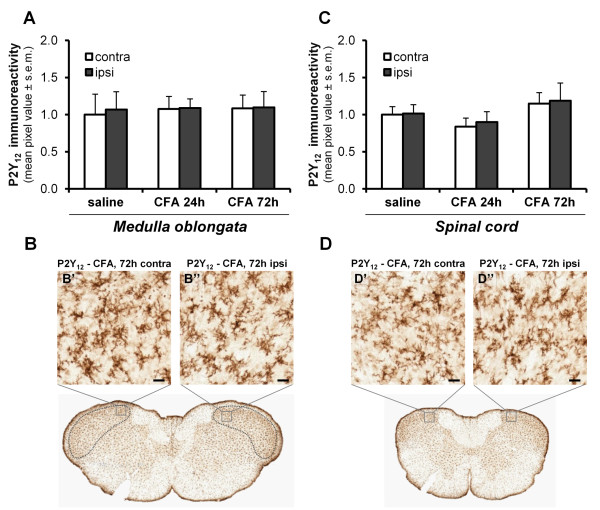
**P2Y_12 _receptor levels in the CNS are not affected by the induction of TMJ inflammation**. (A, C) Densitometric quantification of P2Y_12 _receptor immunoreactivity in both the trigeminal subnucleus caudalis (A) and the cervical dorsal horn (C) revealed no changes between the contralateral (contra) and the ipsilateral (ipsi) sides of inflamed rats. The mean values of pixel intensity have been normalized to the contralateral side of saline injected rats, set to 1.0. Since no significant differences between saline-injected rats at 24 h and 72 h have been observed, data have been pooled together, and shown here as "saline". (B, D) Immunostaining of the P2Y_12 _receptor subtype in the trigeminal subnucleus caudalis (delimited by the dotted line; B-B'') and the cervical dorsal horn (D-D''). Scale bars: 20 μm.

We next evaluated whether P2Y_12 _receptor levels in the spinal trigeminal nucleus were affected by CFA injection into the TMJ or not. Despite the observed upregulation of Iba1 immunoreactivity and the morphological changes observed in microglial cells (see above), no increase in P2Y_12 _receptor immunoreactivity in the ipsilateral side of CFA-injected rats was observed in either the trigeminal subnucleus caudalis (p = 0.957 and p = 0.968 at 24 h and 72 h respectively; n = 7 animals) (Figure 8A-B'') or in the cervical dorsal horn (p = 0.734 and p = 0.923 at 24 h and 72 h respectively; n = 7 animals) (Figure 8C-D''). Taken together, these results demonstrate that the expression of the purinergic P2Y_12 _receptor, which has been implicated in some forms of pain sensations, is not modified in the subacute reaction of CNS glial cells to TMJ inflammation.

## Discussion

In both the CNS and PNS, glial cells have been shown to actively participate in the genesis and maintenance of chronic pain conditions, and might therefore represent innovative targets for the development of new therapeutic approaches to pain management [4,7,9,11,27]. Most of the studies investigating the role of glial cells in inflammatory pain have been performed in animal models of sciatic nerve sensitization, such as the induction of hindpaw inflammation [14,42,58,59] or of joint monoarthritis [41,43], but only limited information on the role of trigeminal SGCs during TMJ inflammation is available [25,31]. Moreover, no characterization of glial cells activation along the whole trigeminal-spinal system is currently available. Thus, in the present study we have examined the response of TG and CNS glial cells following injection of the pro-inflammatory agent CFA in the rat TMJ.

### TG glial and immune cells response to TMJ inflammation

Here, we demonstrate that CFA injection into the TMJ produces a significant increase in GFAP expression in SGCs in the TG, thus confirming the reaction of glial cell to the administration of a pro-algogenic stimulus and validating GFAP as a useful marker of trigeminal sensitization [25,34,35]. The key role of SGCs in the development and maintenance of chronic pain has been demonstrated by their increased expression and release of IL-1β [25], TNFα [18], as well as augmented gap junction-mediated cell coupling [31,60] following nerve injury. All together, these changes are associated with increased excitability of both primary afferents and CNS neurons, leading to the development of hyperalgesia and allodynia [16,33].

We also provide new evidence on the behavior of TG macrophages under inflammatory conditions. In particular, following induction of TMJ inflammation, a strong upregulation of ED1 (a specific marker for activated macrophages) [38] in the ipsilateral TG was found. Because there was no increase in either the number or the average cell size of Iba1^+ ^macrophages, and based on the fact that Iba1 is expressed by both resting and activated microglia/macrophages [61], we conclude that CFA injection does not trigger macrophages infiltration into the trigeminal perineuronal regions from the bloodstream, but rather modulates the activation state of resident immune cells. These cells are, in turn, involved in the development of TG sensitization following TMJ inflammation. This is at variance from previous reports indicating an increase in ED1 immunoreactivity in the DRG following CFA-induced inflammation of the footpad or of the knee joint [37,62] which the authors attributed to macrophage infiltration. Although the total number of immune cells was not evaluated in these studies, and therefore activation of resident cells cannot be excluded, it may well be that the responses evoked in DRG and TG by the same inflammatory event are different, suggesting high regional and time specificity.

### CNS glial cells response to TMJ inflammation

Microglial cells, the resident macrophagic population in the CNS, have been crucially implicated in the initiation and modulation of certain chronic pain states [63]. For instance, in various neuropathic pain models, activated microglia was shown to release proinflammatory cytokines and other substances that facilitate pain transmission [64,65]. Moreover, several drugs acting as glial cell inhibitors (e.g., propentofylline, pentoxifylline and minocyclin) eventually suppress the development of neuropathic pain by decreasing both microglial activation and cytokine release [12,13].

Here we report for the first time that injection of CFA into the TMJ induces a significant ipsilateral activation of microglial cells, both in the dorsal laminae of the trigeminal subnucleus caudalis in the medulla oblongata and in the dorsal horn of the cervical spinal cord, which are the regions receiving the mandibular fibers of the trigeminal nerve [44], and represent key stations for the integration of temporomandibular painful sensations.

Our data are in agreement with previous papers indicating an increased expression of microglial cell markers in the lumbar spinal cord, associated with the appearance of cells having an activated morphology, in CFA-induced ankle or tibio-tarsal joint monoarthritis in rats [41,43]. This confirms that the induction of deep articular inflammation represents a potent trigger for CNS microglial activation. On the other hand, conflicting results have been obtained following induction of subcutaneous hindpaw inflammation by CFA [14,42,58]. Overall, the current hypothesis is that other inflammatory substances rather than CFA, like formalin or zymosan, can activate spinal cord microglia following their hindpaw injection, suggesting a strict dependence of microglia recruitment from both the inflammatory stimulus and the injection site.

We also examined astrocyte activation in the brainstem following TMJ inflammation. At variance from previous reports on sciatic nerve inflammation [42,43], we did not detect any evidence of astroglial activation. This divergence could be due to the fact that the latter studies showed astroglial activation occurred at later time points (11 and 14 days) following the inflammatory insult, thus suggesting that astrocytes are not involved in the first sub-acute phases of tissue sensitization.

### No evidence for upregulation of the P2Y_12 _receptor subtype in the sub-acute phases following TMJ inflammation

The purinergic P2Y_12 _receptor is a Gi-coupled metabotropic receptor specifically activated by extracellular ADP [52] that has been shown to control the chemotaxis of CNS microglial cells in response to nucleotides [56,66]. Moreover, microglial P2Y_12 _receptors have been demonstrated to increase at both the mRNA and protein level in the ipsilateral lumbar spinal cord following nerve injury in rats [54,55], and both the pharmacological and biotechnological blockade of this receptor prevented the development of neuropathic pain hypersensitivity [54,55]. Furthermore, P2Y_12 _knock-out mice displayed deficiency in the development of tactile allodynia following nerve injury, despite normal basal mechanical sensitivity [55]. Therefore the P2Y_12 _receptor can be considered as a key player in controlling microglia-associated development of neuropathic pain, thus representing a possible therapeutic target for treating chronic pain disorders. For these reasons we checked for its possible modulation in our inflammatory pain model. By utilizing a custom-made antibody whose specificity was already successfully validated in P2Y_12_-deficient mice [56], no P2Y_12 _immunostaining was detected on TG sections, although we have previously detected P2Y_12 _mRNA in mouse TG [50]. Interestingly, staining for the P2Y_12 _receptor was observed at the boundary between the trigeminal nerve root and the CNS, and in Iba1^+ ^cells of the brainstem. This observation correlates with previous reports indicating that, unlike other extracellular nucleotide receptors, such as the P2X_4_, P2X_7_, and P2Y_6 _receptor subtypes [53,67], P2Y_12 _receptor expression is restricted to CNS microglia [56,68]. Quite surprisingly, we detected no changes in microglial P2Y_12 _receptor levels after the injection of CFA in the TMJ, suggesting that this receptor is not up-regulated during the first phases of tissue hypersensitivity following an inflammatory stimulus. It is worth mentioning that in a different model of inflammatory pain (i.e., the injection of CFA in the hindpaw) the intraperitoneal administration of the P2Y_12 _receptor antagonist 2,2-dimethyl-propionic acid 3-(2-chloro-6-methylaminopurin-9-yl)-2-(2,2-dimethyl-propionyloxymethyl)-propylester (MRS2395) significantly alleviated the mechanical hypersensitivity [69], but authors did not evaluated the possible changes in P2Y_12 _receptor expression in their experimental model. Therefore, it may well be that, despite the lack of detectable receptor up-regulation, P2Y_12 _receptor targeting might be beneficial also for treating other chronic inflammatory pain states involving the sensitization of the trigeminal-spinal system, as in our experimental model.

## Conclusions

In the present study we have characterized glial cell reaction in the nervous system in a rat model of TMJ inflammation. Specifically, we have shown that CFA injection is associated with activation of SGCs and macrophages in the TG, as well as of microglial cells in the spinal trigeminal nucleus, with no signs of reactive astrogliosis. Therefore, our results clearly demonstrate a correlation between the activation of both TG and CNS glial cells and inflammatory pain. Additional studies are now needed in order to demonstrate that the observed glial activation in one or both sites plays a direct role in either the initiation or the maintenance of the pain behavior, and to suggest glial cells as possible innovative pharmacological targets for treating trigeminal-associated pain.

## Methods

### Animals

Experiments were performed on adult male Sprague-Dawley rats (200 - 250 g; Charles River Lab, Calco, Milan, Italy). Animals were housed under controlled conditions (temperature 22 ± 2°C; relative humidity 50 ± 10%; artificial light 12 h light/dark cycle, lights on at 7 AM). All animals had access to both distilled water and standard diet ad libitum. The study has been approved by the Council of the Department of Pharmacological Sciences of the Università degli Studi di Milano, Milan, Italy, and was carried out in accordance with National and European regulations regarding the protection of animals used for experimental and other scientific purposes (D.M. 116192; 86/609/EEC), as well as following the ethical guidelines of the International Association for the Study of Pain (IASP) [70].

### Induction of TMJ inflammation

The sub-chronic TMJ inflammation was induced by injecting 50 μl of CFA (Sigma, Milan, Italy) oil/saline (1:1) emulsion into the left TMJ capsule, under isoflurane anesthesia. Control rats were injected with saline (0.9% NaCl). The TMJ capsule was identified by palpating the zygomatic arch and condyle, and the injection was delivered by advancing a 27-gauge needle medioanteriorly through the skin immediately below to the posteroinferior border of the zygomatic arch until it entered into the joint capsule [71]. Then, CFA or saline was slowly injected over two minutes.

### Behavioral test

Mechanical allodynia was measured by a previously described protocol with some minor modifications [72]. Unrestrained rats were trained to stay in position and to be probed with von Frey filaments (North Coast Medical, Morgan Hill, CA, USA) at least one week before the injection of CFA. The left and right orofacial skin regions, near to the center of the vibrissa pad, were tested. An ascending series of von Frey filaments was used. The starting filaments corresponded to log unit 4.31 (force: 2 grams) and 3.22 (force: 0.16 grams) for control and inflamed animals, respectively. Each filament was tested five times with an interval of a few seconds. The response threshold was defined as the lowest force required to elicit at least three head withdrawal responses out of five tests. The elapsed time between the applications of a new filament was 2 minutes. All experiments were carried out in a quiet room between the 8.30 AM and 1.00 PM, in order to avoid diurnal variations.

### Measurement of Evans' blue dye extravasation

Evans' blue dye (5 mg/kg, 0.3% solution) was injected into the tail vein [73], 10 minutes before perfusion (see below). Ipsi- and contra-lateral (with respect to the side of CFA injection) TMJs were then dissected, cut into small blocks and incubated overnight at room temperature (RT) in a 7:3 (vol/vol) mixture of acetone and 35.2 mM sodium sulfate on a shaking table. Samples were then centrifuged, the supernatant separated, and dye absorbance determined in a spectrophotometer at 620 nm. Evans' blue dye concentrations, in μg/ml, were extrapolated from a best-fit line calculated from a standard curve, prepared from a series of supernatants extracted from the TMJs of naïve animals, and mixed with serial dilutions of the Evans' blue dye.

### Tissue processing

Twenty-four and 72 hours after CFA injection, rats were anesthetized with intraperitoneal injection of 400 mg/kg chloral hydrate and transcardially perfused with 4% formalin fixative. Both the intact brain and TGs were then excised, postfixed in 4% formalin for 60-90 min, and cryoprotected in 30% sucrose for at least 48 hours. The left and right TG from each animal were embedded together in mounting medium (OCT; Tissue Tek, Sakura Finetek, Zoeterwoude, The Netherlands), and cut longitudinally on a cryostat at 15 μm thickness. Brainstems were separated from the rest of the brain, and marked ventrally on the contralateral side to subsequently identify tissue orientation. Transverse 40 μm thick free-floating sections were then cut on a cryostat.

### Immunohistochemistry

Free-floating brainstem sections, or on-slide TG sections, were incubated for 45 min at RT in PBS containing 10% normal goat serum (Sigma-Aldrich, Milan, Italy) and 0.1% Triton X-100 (Sigma-Aldrich), and then overnight at RT with the following primary antibodies: rabbit anti-glial fibrillary acidic protein (GFAP, 1:600; Dako, Milan, Italy), mouse anti-NeuN (1:500; Millipore, Vimodrone, Italy), rabbit anti-ionized calcium binding adaptor molecule 1 (Iba1, 1:800; Biocare Medical, Space Import-Export, Milan, Italy), rabbit anti-P2Y_12 _receptor polyclonal antiserum (1:1,500; a generous gift by Prof. David Julius, University of California San Francisco, CA, USA), and mouse anti-ED1 (1:200; Serotec, Space Import-Export).

For fluorescence analysis, sections were then rinsed three times with PBS, and incubated for 1 h at RT with goat anti-rabbit and goat anti-mouse secondary antibodies conjugated to AlexaFluor^®^488 or AlexaFluor^®^555 fluorochromes (1:600; Molecular Probes, Invitrogen, Milan, Italy). Nuclei were subsequently labeled with the fluorescent dye Hoechst 33258 (1:10,000 in PBS; Molecular Probes). Slides were finally washed, mounted with Fluorescent Mounting Medium (Dako), and examined with a laser scanning confocal microscope (LSM 510; Zeiss, Jena, Germany). Images were acquired and analyzed using the LSM Image Browser software (Zeiss).

For light microscopy, sections were incubated for 1 h at RT with an anti-rabbit biotinylated secondary antibody (1:500; PerkinElmer, Monza, Italy), and then with a horseradish peroxidase (HRP)-conjugated streptavidin (1:400, 45 min at RT; PerkinElmer). To visualize the formation of the antibody-antigen complex, the nickel-3,3'-diaminobenzidine (Sigma-Aldrich) protocol was used. Sections were mounted with DPX (Sigma-Aldrich), and analyzed with an inverted microscope (Axiovert 200; Zeiss) equipped with a color CCD camera (AxioCam HRc; Zeiss), connected to a PC computer equipped with the software Axiovision (Zeiss).

Non-specific staining was evaluated on sections where the primary antibodies were omitted from the staining procedure. All antibodies were diluted in PBS containing 0.1% Triton X-100 and 1% normal goat serum.

### Quantifications and data analysis

Quantitative analysis of immunopositive cells in the TG and in the brainstem was performed by using the NIH Image-J software on digital images of immunolabeled sections. To avoid variability in the staining procedure, all the sections to be compared were processed together, and images were acquired under the same exposure conditions. Two sections for each TG or brainstem and for each labeling antibody were analyzed.

The number of Iba1^+ ^macrophages in TG was measured on sections captured at 10× magnification. A stack of all acquired images was created, and the threshold for analysis was set to a level that included all Iba1-immunopositive cells, but not the lighter pixels of the background. The perineuronal regions of the V1, V2 and V3 division were outlined, and both the number and the average size of Iba1^+ ^cells were then measured. The number of positive cells has been normalized to the outlined area of measurement. The number of ED1^+ ^macrophages in TG was estimated by counting the number of immunopositive cells at 40× magnification.

ED1 immunoreactivity in the TG, and GFAP, Iba1 and P2Y_12 _receptor immunoreactivity in the brainstem was assessed by densitometric analysis. A digital image of the immunolabeled sections was acquired at 20× or 10× magnification for TG or brainstem, respectively, and the threshold for analysis of positive staining was set as described above. The mean values of pixel intensity were then automatically counted by using the NIH Image-J software. For GFAP-, Iba1- and P2Y_12_- receptor immunostaining, the mean pixel intensity values were expressed as fold increase compared to the contralateral side of saline injected animals set to 1.0. The anatomical structures in the brainstem and spinal cord were identified with reference to a rat brain atlas [74].

All results are expressed as mean ± s.e.m. of at least three independent experiments. Statistical significance between groups was derived from one-way ANOVA followed by Scheffe's analysis, performed with the SPSS software. Three degrees of significance were considered: p < 0.05 (*), p < 0.01 (**), p < 0.001 (***).

## Abbreviations

CFA: Complete Freund's Adjuvant; CNS: central nervous system; DRG: dorsal root ganglia; Iba1: ionized calcium binding adaptor molecule 1; GFAP: glial fibrillary acidic protein; IL: interleukin; PNS: peripheral nervous system; RT: room temperature; SGCs: satellite glial cells; TG: trigeminal ganglion; TMJ: temporomandibular joint; TNFα: tumor necrosis factor-α.

## Competing interests

The authors declare that they have no competing interests.

## Authors' contributions

GV contributed to the study design, performed all the experiments, acquired and analyzed data and drafted the manuscript. MZ contributed to in vivo studies and to the acquisition and analysis of data. GM contributed to in vivo studies and to the acquisition and analysis of data

SC participated to the study design, supervised the experimental procedures, and drafted the manuscript. LJ and PTO contributed to the study design and drafted the manuscript. MPA participated to the study design, supervised the experimental procedures and drafted the manuscript. All authors read and approved the final manuscript.
